# Non-invasive determination of murine placental and foetal functional parameters with multispectral optoacoustic tomography

**DOI:** 10.1038/s41377-019-0181-7

**Published:** 2019-08-14

**Authors:** Kausik Basak, Xosé Luís Deán-Ben, Sven Gottschalk, Michael Reiss, Daniel Razansky

**Affiliations:** 1 Faculty of Medicine, Technical University Munich, Munich, Germany; 2 Institute for Biological and Medical Imaging, Helmholtz Center Munich, Neuherberg, Germany; 3 Faculty of Medicine and Institute of Pharmacology and Toxicology, University of Zurich, Zurich, Switzerland; 4 Institute for Biomedical Engineering and Department of Information Technology and Electrical Engineering, ETH Zurich, Zurich, Switzerland; 5 Present Address: Kausik Basak, Institute of Advanced Studies and Research, JIS University, Kolkata, West Bengal India

**Keywords:** Photoacoustics, Biophotonics, Imaging and sensing

## Abstract

Despite the importance of placental function in embryonic development, it remains poorly understood and challenging to characterize, primarily due to the lack of non-invasive imaging tools capable of monitoring placental and foetal oxygenation and perfusion parameters during pregnancy. We developed an optoacoustic tomography approach for real-time imaging through entire ~4 cm cross-sections of pregnant mice. Functional changes in both maternal and embryo regions were studied at different gestation days when subjected to an oxygen breathing challenge and perfusion with indocyanine green. Structural phenotyping of the cross-sectional scans highlighted different internal organs, whereas multi-wavelength acquisitions enabled non-invasive label-free spectroscopic assessment of blood-oxygenation parameters in foeto-placental regions, rendering a strong correlation with the amount of oxygen administered. Likewise, the placental function in protecting the embryo from extrinsically administered agents was substantiated. The proposed methodology may potentially further serve as a probing mechanism to appraise embryo development during pregnancy in the clinical setting.

## Optoacoustic imaging: Monitoring placental and fetal health

A non-invasive imaging tool that reveals blood oxygen levels and perfusion in mice placentas and fetuses has potential applications for monitoring preeclampsia and other pregnancy disorders. Optoacoustic (OA) imaging involves sending laser pulses into biological tissue. The light absorption results in emission of ultrasonic waves, which are detected and used to build up images representing physiological factors such as oxygen levels. Daniel Razansky at the University of Zurich and ETH Zurich, Switzerland, and co-workers used OA imaging to non-invasively monitor placental and fetal function in pregnant mice. The team administered near-infrared contrast agent and alternate doses of 20 and 100% oxygen to the mice via breathing apparatus and monitored the resulting blood oxygenation and perfusion parameters. The images provided unprecedented detail showing how the placenta protects the embryo from excessive oxygen and extrinsically administered agents.

## Introduction

Medical history has witnessed multiple complications during pregnancy and childbirth due to impaired oxygen transfer across the foeto-maternal vascular interface, resulting in pregnancy-induced hypertensive disorders (PIHD), including preeclampsia, with possible fatal maternal and/or foetal effects. Anomalous placental development and lack of oxygen perfusion through the placental-vascular interface can lead to hypoxic and ischaemic attacks. According to a survey by the World Health Organization, ~830 women die every day across the world due to complications related to pregnancy and childbirth, among which ~14% are attributable to PIHD^1^. Moreover, placental insufficiency and maternal chronic hypoxia hinder oxygen delivery to the foetus, leading to several consequences, including intrauterine growth restriction (IUGR) and foetal death^2^. However, despite the importance of the placental function in embryonic development, it is still not fully understood and is challenging to characterize in vivo. Therefore, the emergence of new insights in placental insufficiency and oxygenation along with changes in maternal and foetal functional parameters are of high importance and can help in the prevention of pathological cases via periodic monitoring of anatomical and functional attributes during development.

With technological advancements in imaging methods, several probing mechanisms, such as Doppler ultrasound (US)^3^, X-ray computed tomography (CT)^4^, and blood oxygen level-dependent magnetic resonance imaging (BOLD MRI)^5,6^, have been shown to have considerable potential for better understanding of embryonic development in pre-clinical research and in clinical practice. Doppler-US provides high spatial resolution for structural phenotyping but lacks the capability of measuring placental oxygen saturation. X-ray CT provides a weak soft-tissue contrast, and its radiation exposure limits longitudinal studies, particularly in obstetrics. Additional limitations stem from the need for exogenous contrast agents and the incapability of real-time imaging of the embryo. BOLD MRI can provide high-resolution anatomical images along with estimates of changes in placental oxygen saturation. However, its clinical application is limited by the high instrumentation costs associated with microscopic level resolution and the inability to operate at the bedside. Overall, none of the clinically established imaging modalities can offer real-time readings of placental and foetal oxygenation and perfusion parameters.

Optoacoustic (OA) imaging emerged in the early 2000s as a novel non-invasive imaging method harnessing the advantages of optical and US imaging modalities to provide high-contrast characteristic responses of functional and molecular attributes without sacrificing resolution (for depths of millimetres to centimetres) in highly optically scattering biological tissues^7,8^. After several years of continuous technological developments, the use of OA in pre-clinical research and, more recently, in clinical studies has widely spread, with applications including characterization of cerebral hemodynamic responses upon external stimuli^9^, multi-scale whole-body imaging of small animals^10,11^, cell tracking^12,13^, assessments of tumour growth and progression^14,15^ and visualization of cardiovascular dynamics^16^. OA has been shown to complement or enhance the hemodynamic readings provided by Doppler US^17^ or functional MRI^18^. Particularly, five-dimensional OA tomography can simultaneously measure multiple hemodynamic parameters in real time^19^ that have remained inaccessible with other modalities. In addition, OA has successfully demonstrated its potential in delivering highly specific information from targeted and activatable probes^20^ as well as from genetic labels^21,22^. Moreover, OA imaging has shown significant ability to detect retinal neovascularization in larger animals^23^, which can be further exploited in the investigation of retrolental fibroplasia (RLF) or retinopathy of prematurity (ROP), a major cause of blindness in premature cases that originates from factors such as neovascularization, high oxygen exposure to preterm babies^24,25^ among others. OA structural phenotyping of mammalian embryos has been shown to be feasible by either imaging mice embryos ex vivo^26^ or using relatively slow scanning-based systems^27,28^. More recent studies have focused on monitoring placental and foetal oxygenation at different developmental stages with real-time OA systems based on linear arrays^2,7,29^. However, these systems are strongly affected by so-called limited-view effects and hence offer sub-optimal imaging performance and very limited quantification capabilities^30^. Significant limitations yet remain in terms of inadequate depth penetration and lack of a high-resolution anatomical lay-out of whole cross-sectional areas, thereby encumbering its application in the clinical domain. More importantly, the spectroscopic imaging paradigm has not been fully explored in simultaneous monitoring and comparative evaluation of changes in oxyhaemoglobin (HbO) and deoxyhaemoglobin (HbR) from maternal to foeto-placental areas.

This work focuses on the study of the capabilities of a self-developed ring-shaped optoacoustic tomography (ROAT) system based on an array of cylindrically focused transducers for in vivo and non-invasive embryonic imaging in pregnant mice. Particular emphasis is placed on functional analysis of the spatio-temporal distribution of hemodynamic parameters, such as HbO, HbR and oxygen saturation (SO_2_), in different areas (maternal peripheral artery, placenta and embryo region) of tomographic scans. The system can provide cross-sectional images over a large area (~40 mm across) with a high (~200 µm) spatial resolution^31^, providing both structural and functional data from pregnant mice at gestation days E14 and E19.

## Results

**Fig. 1:**
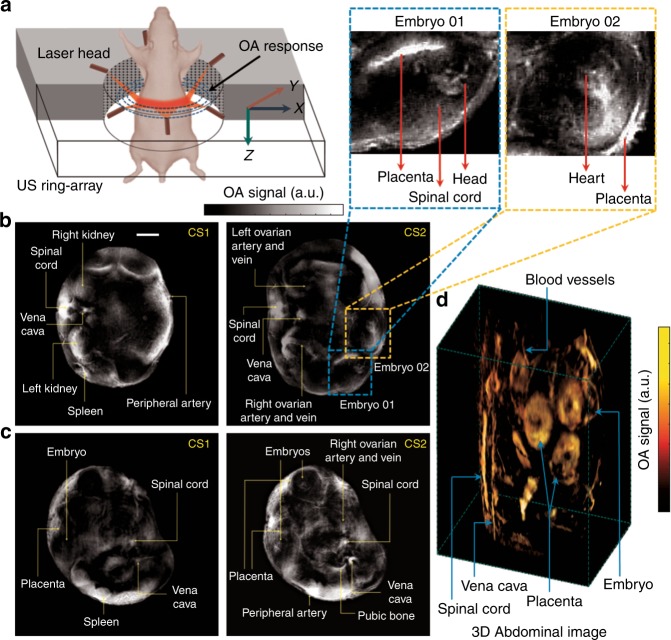
In vivo whole-body imaging of pregnant mice at gestations days E14 and E19. **a** Schematic of the ring-shaped optoacoustic tomography (ROAT) system. **b** In vivo tomographic scans of the abdominal cavity of an E19 pregnant mouse at two different cross-sections (CS1 and CS2): anatomical lay-out highlighting the different internal organs of the mother mouse along with embryos, labelled on the zoomed-in sections. **c** Similar cross-sections (CS1 and CS2) from an E14 pregnant mouse with anatomical phenotyping of various internal organs in the abdominal region. The scale bars for both (**b**, **c**) are 5 mm. **d** Three-dimensional abdominal image of an E14 mouse acquired with spiral volumetric optoacoustic tomography (SVOT)^10^

The developed small-animal imaging scanner (see Fig. 1a and the “Methods” for details) enables in vivo, non-invasive and real-time probing with fast-scanning and high-resolution imaging of pregnant mice. The selected range of the near infra-red (NIR) regime (700–1064 nm) maximizes light penetration in mammalian tissues. Anatomical data were acquired around the abdominal cavity to locate embryos and different internal organs of mother mice. Reconstructed and processed images at 1064 nm of illumination are shown in Fig. 1b, c with structural phenotyping of different cross-sections at E19 and E14 gestation days, respectively. In the largest pregnant mouse (Fig. 1b: CS2 - E19, ~40 mm diameter), four embryos are observed in a single cross-section. The characteristic response of the OA signal from different internal organs and embryos facilitates label-free annotations. Different internal organs within the abdominal cavity of the mother mice, such as the spinal cord, vena cava, ovarian artery and vein, pubic bone, spleen, kidney and so on, are labelled in Fig. 1b, c. Likewise, embryonic parts, such as the placenta, head, spinal cord, and heart, can be accurately observed in the E19 cross-sectional scan and are elucidated in the zoomed-in sections. We further performed a whole-body spiral volumetric tomography (SVOT) scan of the mouse, as previously reported^10^ (see Supplementary Fig. 2). A fly-through movie of a vertical translation scan of an E14 pregnant mouse is presented in Supplementary Video 1. The SVOT method does not allow for real-time tracking of fast-dynamic events at the whole-body level but enables an easier anatomical interpretation of the embryo locations in the different cross-sections (Fig. 1d).

**Fig. 2:**
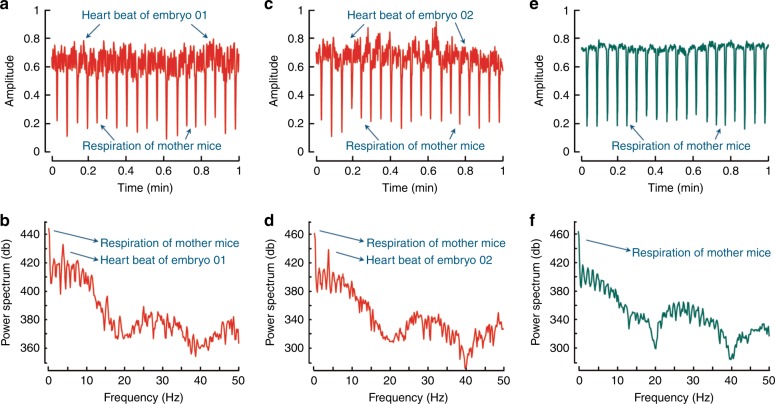
Analyses of the respiratory motion of an E19 mother mouse and embryo heartbeat. Time activity curves of the OA signal acquired from two different embryo hearts are shown in (**a**, **c**) with their corresponding frequency spectra plotted in (**b**, **d**). No spatio-temporal averaging was applied for calculating the plots. Respiration of mother mice can be approximated at 0.7 Hz, whereas the embryo heartbeat falls in the 4–5 Hz range. The labelled frequency peaks embody the heartbeats of embryos and respiration of mother mice. **e** Changes in the OA signal at the maternal peripheral artery capture only its respiratory motion, which is also prominent in its corresponding Fourier spectrum in (**f**)

The fast acquisition rate of the ROAT system allows both the respiratory motion of mother mice and the heartbeat of the embryo to be tracked, which are further analysed in Fig. 2. A fly-through movie of reconstructed tomographic scans of an E19 mouse is presented in Supplementary Video 2. By tracking the temporal changes in signals at the locations of the embryo hearts (embryo 1 and 2 in Fig. 1b) and the peripheral artery of the mother mouse, the embryo heartbeat and respiration of the mother mouse were obtained (Fig. 2a, c, e). The heartbeats of embryos were comparatively smaller in pitch and higher in frequency than the respiratory motion of mother mice. The Fourier spectra of these time domain signals are shown in Fig. 2b, d, f. The first two peaks in the power spectrum density plots (Fig. 2b, d) represent the respiration of mother mice (~0.7 Hz) and embryo heartbeat (~4.5 Hz). The time activity curve at the peripheral artery of mother mice (Fig. 2e) signifies absence of the embryo’s heartbeat, which can additionally be substantiated from its equivalent Fourier domain spectrum (Fig. 2f).

**Fig. 3:**
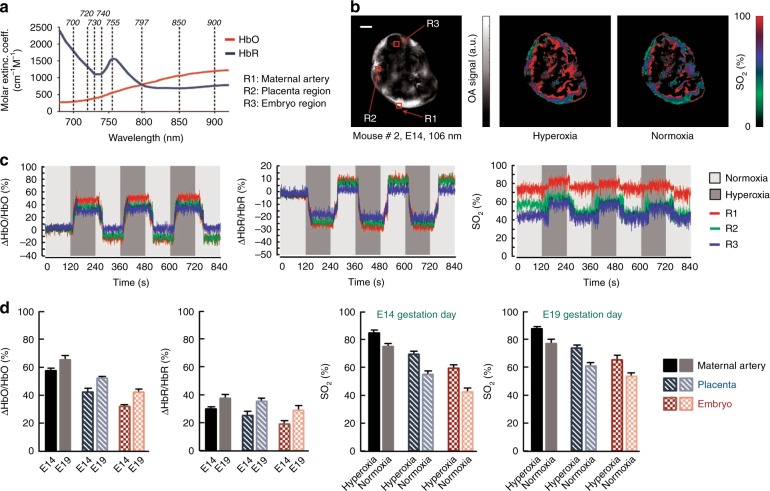
Multispectral imaging of the functional characteristics of a maternal mouse and embryo under hyperoxia and normoxia conditions. **a** Molar extinction spectrum of HbO and HbR in the near infra-red spectrum. The multispectral data sets were acquired at the five indicated wavelengths. **b** Representative cross-sectional ROAT reconstruction at 1064 nm along with the spectrally unmixed SO_2_ distributions under hyperoxic and normoxic conditions. The scale bar corresponds to 5 mm. Comparative analyses of the HbO, HbR and SO_2_ variations were performed in maternal (R1), placenta (R2) and embryo (R3) regions. **c** Time course of changes in HbO, HbR and SO_2_ are plotted for the three regions. **d** Comparative evaluation of the relative hemodynamic changes during the breathing gas challenge at different locations corresponding to maternal artery, placenta and embryo regions for different groups of mice at gestation days E14 and E19

**Fig. 4:**
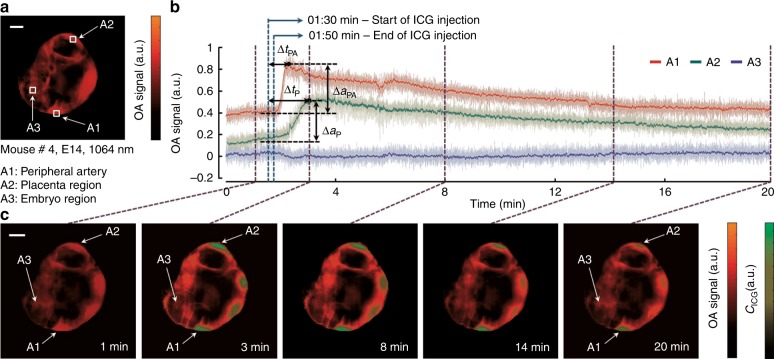
Study of the ICG kinetics in an E14 mouse. **a** Reference cross-sectional ROAT image of a mouse at 1064 nm in which the three evaluated areas are labelled. **b** Time lapse OA signal traces acquired at 800 nm. Tail-vein injection of ICG was initiated at *t* = 90 s and lasted for 20 s. Raw signals are presented in the light shaded colour form (in background), whereas the deep colour foreground signals are computed using a moving average (10 point) filter. Note that although there is a delay observed between the appearance of ICG in the placenta (Δt_P_) and the peripheral artery of the mother mice (Δt_PA_), the change in the OA signal is significantly higher in the peripheral artery location (Δa_PA_) compared with the placenta (Δa_P_) due to stronger ICG perfusion. Likewise, no change in OA signal was observed in the embryo region. (**c**) Spectrally unmixed ICG distribution (in green) at different time points prior and post injection. Scale bars are 5 mm

A study of the changes in the functional parameters obtained from the multispectral images was carried out in two phases, namely, a breathing gas challenge (Fig. 3) and ICG perfusion (Fig. 4). Prior to these challenges, single wavelength scans were acquired at 1064 nm to locate a suitable cross-section in which both the embryo and placenta could be accurately observed. Figure 3 elaborates on the functional characteristic responses in an E14 pregnant mouse when subjected to an oxygen challenge. The molar extinction coefficients of blood chromophores (HbO and HbR) at NIR wavelengths are provided in Fig. 3a. The anatomical scan with excitation at 1064 nm is shown in Fig. 3b, where different regions of interest (ROIs), e.g., R1, R2 and R3, located at the peripheral artery of the mother mouse, placenta and embryo region respectively, are indicated. Maps of SO_2_ (Fig. 3b) during hyperoxic and normoxic exposure of pregnant mice were rendered from the spectrally unmixed data. A subsequent response in SO_2_ from hyperoxic to normoxic conditions is clearly observed. The spectrally unmixed images showing the HbO and HbR distributions on whole cross-sections of pregnant mice scans following breathing gas stimulation are shown in Supplementary Fig. 3. The characteristic responses in the unmixed images were spatially averaged (5 × 5 pixels) within the indicated ROIs to compute the changes in the HbO and HbR levels. The percentile changes in HbO and HbR at these locations are given in Fig. 3c, in which a distinctive change can be observed during switching from normoxia to hyperoxia, and vice versa. The baseline values were calculated as the average of the first 20 frames (in normoxia). The changes in HbR appear more in negative percentiles. From the HbO time profiles, it is apparent that the relative changes in HbO in a peripheral artery (~50–60%) are higher than in the placenta (~35–40%) and embryo (~25–30%) regions at hyperoxia. Likewise, a higher HbR can be observed in the peripheral artery compared to the placenta and embryo, justifying the higher change in perfusion of these blood chromophores in the maternal vasculature than in the placental and embryonic vasculature during an external oxygen challenge. Similar average values of the changes in HbO and HbR for pregnant mice at different gestation days (E14 and E19: 2 mice each) were consistently measured, although small percentile differences between E14 and E19 were observed (Fig. 3d). Furthermore, the SO_2_ map reflects a greater change in the maternal artery compared to the placental region and embryo at 100% O_2_ exposure. Figure 3d highlights SO_2_ recordings (average value of different ROIs) at different gestation days (E14 and E19) in which no such significant change in SO_2_ levels could be observed. The measured changes of SO_2_ in the maternal artery and placenta are in close accordance with the theoretical values, as previously reported^7^. Although a quantitative measurement of SO_2_ in the entire cross-sections is challenging, it appears that the maternal artery consistently exhibits higher signal changes compared with the placenta and embryo regions.

Additionally, we explored the perfusion of ICG from the maternal region to the foeto-placental area after a tail-vein injection into an E14 mouse. Such observations may enable the understanding of perfusion of ICG through the placental barrier and substantiate its function in protecting foetuses from external stimuli. Again, an optimal cross-section containing several embryos was first identified using 1064 nm laser illumination (Fig. 4a). A sequence of images during the ICG injection were then acquired on the same section at 800 nm laser illumination (see the “Methods” section). Figure 4b shows three-time profiles of the OA signals over the entire acquisition window. These profiles illustrate ICG perfusion at three different locations, namely, the peripheral artery of the mother mouse (A1), the placenta (A2) and the embryo region (A3). These areas are noted on the anatomical cross-section shown in Fig. 4a. The ICG injection was initiated at 90 s after the start of the acquisition and lasted for 20 s. A significant rise in optical contrast can be observed in areas A1 and A2 due to strong ICG perfusion. At the same time, the OA signal remained stable in the embryonic area, confirming the functionality of the placental barrier in hindering the perfusion of ICG to the embryo. Although a small delay exists between the time of injection and the appearance of the contrast agent in the peripheral artery (Δ*t*_PA_) and placenta (Δ*t*_P_), where Δ*t*_PA_<Δ*t*_P_, the changes in the OA signal are significantly higher in the peripheral artery (Δ*a*_PA_) compared to the placenta (Δ*a*_P_) due to higher ICG perfusion. The lower panel (Fig. 4c) shows subsequent images of the imaged cross-section before and after the ICG injection. The change in the distribution of the contrast agent over time can be readily determined from these images as sharp variations of the unmixed signal component (in green) in the aforementioned locations. At 3 and 8 min after the injection, the high contrast in A1 and A2 depicts strong ICG perfusion. Later, the contrast approached a plateau owing to the reduction in ICG (images at 14 and 20 min). However, no change in contrast was identified in the embryo region throughout the acquisition.

## Discussion

This work demonstrates that ROAT is suitable for monitoring placental and embryonic function during pregnancy in rodents. Imaging at a wavelength of 1064 nm facilitated accurate labelling and identification of internal organs within the abdominal cavity of pregnant mice. Pulsations associated with the heartbeat of the embryo and the respiration of the maternal mouse were further observed with a high spatio-temporal resolution. More subtle anatomical structures of the embryo may be resolved by using US arrays with a broader bandwidth, thus attaining a better spatial resolution. In addition, multi-wavelength image acquisition at 700, 730, 755, 800 and 850 nm enabled the analysis of functional attributes, such as HbO, HbR and SO_2_, in mice subjected to an oxygen challenge with periodic 100% and 20% O_2_ exposure. Although the observed changes in placental and embryonic oxygen saturation were lower than in the mother mouse, the changes in functional parameters are conclusive for a change in foetal blood oxygenation due to hyperoxia, which further confirms the reserve oxygen capacity of the placenta in accidental changes in oxygen exposure to the maternal body^2^. The large capillary network in the placenta (during the course of gestation) may preserve placental oxygen for a period of time, so that relative changes in oxygen exposure may have a lower effect in the placenta and hence in the embryo. Similar studies were performed to assess placental and foetal oxygenation using BOLD MRI in different animal models, where a subsequent change in foetal blood oxygenation was reported during hypoxia of the maternal animal^32,33^. A comparison of OA hemodynamic readings with standard BOLD MRI signals was conducted in a previous study^18^ where a good correlation was found in the hemodynamic parameters of tumour vasculature in mice in vivo. Functional studies with respect to the change in oxygen concentration at normoxia and hypoxia have also been performed^2^, in which a gradual decrement in oxygen saturation in the placental and skin regions of the mother mouse was shown while changing the oxygen exposure from hyperoxia to hypoxia. Our study is the first to extend pre-clinical placental and embryonic research to the whole-body scale in pregnant mice to characterize various anatomical features and subsequently perform a functional study in a longitudinal manner. To ascertain the functional attributes and efficacy of the ROAT approach, an oxygen challenge (hyperoxia) was induced in pregnant mice. The change in the blood oxygenation parameters (HbO, HbR and SO_2_) while changing the oxygen exposure from 100% to 20% also depicts the nature of their alterations, substantiating the standard rule of change in blood-oxygenation parameters. Considering the established correlation between the OA response to blood oxygenation and Doppler flow^34^, our functional responses are in good agreement with the previously reported data, substantiating the efficacy of the ROAT imaging instrumentation for non-invasive studies with an unprecedented spatio-temporal resolution and field of view. We can therefore state that ROAT is a potentially viable imaging paradigm to monitor changes in the functional attributes of foetal and placental regions, which may help to overcome complications during gestation periods. For example, RLF and other pathological changes in the retina are known to be induced in the foetus by hyperoxic conditions^35^. In this context, the designed system and methodology can also provide significant input into the investigation of the pathologic conditions related to high oxygen exposure (hyperoxia) in premature infants, causing RLF in resource-limited clinical settings. Moreover, inadequate O_2_ perfusion through the placenta can directly cause placental insufficiency and IUGR. Early symptoms of trivial embryo development and onset of placental insufficiency can potentially be detected by in vivo monitoring of the placental oxygenation. In this context, ROAT can significantly reduce the risk of anomalous placental function leading to abnormal embryo development and often foetal death.

The functionality of the placental barrier in protecting the embryo from external stimuli was observed in an additional experiment in which the perfusion of ICG through the placental barrier was dynamically monitored. Although this experiment was aimed at studying the functional behaviour of the placenta, it is important to emphasise the fact that only a qualitative assessment of the ICG distribution was performed. The linear unmixing method used in this work enables only qualitative temporal mapping of ICG at deeper locations. Quantitative estimations are challenging in this situation due to alterations in the spectral signature of OA signals owing to wavelength-dependent light attenuation in deep tissues^36^, which to quantification errors in approximating the bio-distribution of ICG in deep vascular networks. Although other spectroscopic algorithms based on blind unmixing or statistical approaches have been shown to enhance the sensitivity of detecting specific chromophores^37^ and eigenspectra analysis has been employed to mitigate wavelength-dependent light fluence variations^38^, quantification inaccuracies generally remain in images. Likewise, the sensitivity to other external stimuli must be studied to obtain a detailed characteristic response of the foeto-placental function.

The demonstrated good performance of the ROAT imaging system in providing both anatomical and functional information about maternal and foetal bodies in real-time and in a non-invasive manner further validates its possible clinical application in monitoring foeto-placental health through measurement of different blood-oxygenation parameters. In this regard, hand-held OA scanners based on the same type of concave arrays of cylindrically focused transducers have been shown to offer promising prospects for characterizing human tissues at centimetre-scale depths^39^. It may then be feasible to assess the anatomical and functional attributes of the human placenta as well as to monitor embryonic development in women suffering from pathological placental insufficiencies. It is important to take into account that minor changes in oxygen saturation with respect to normoxia are expected in the clinical scenario, and the sensitivity of the system must be evaluated in such cases. However, monitoring the human embryo is expected to be hampered by a limited penetration of photons even for NIR laser sources. To facilitate clinical translation, the light delivery approach must first be optimized along with the frequency bandwidth, number of elements and arrangement of such transducer arrays. This optimization would allow the pregnancy stages in which the new imaging approach may be applicable in clinics to be determined. It is also important to highlight that ICG is an FDA-approved contrast agent at the concentrations injected in our study^40^. Therefore, assessment of the functionality of the placenta as a barrier against external agents may also be possible in humans. The reduced changes in oxygenation compared with the maternal peripheral artery, as reflected by the experiments with a breathing gas challenge, corroborate two important facts: placental insufficiency due to maternal oxygen challenge and preservation of placental oxygen even in the challenging environment. Additionally, the performed ICG perfusion experiments substantiated the functionality of the placental barrier in protecting the embryo from external stimuli. Thus, the new imaging approach can potentially be leveraged in periodic monitoring of maternal and foetal health and also in clinical applications to study and aid pregnancy-related abnormalities.

## Methods

### Ring-shaped OA tomography system

A schematic description of the whole-body ROAT is presented in Fig. 1a with its detailed outline available in Supplementary Fig. 1. The illumination system comprises a dual-output (output1: 1064 nm and output2: 700–900 nm) custom-made optical parametric oscillator-based laser (Innolas Laser GmbH, Krailling, Germany) capable of generating pulses with a repetition frequency (PRF) up to 100 Hz and <10 ns pulse duration with energy approaching 30 mJ. Imaging at 1064 nm was performed for anatomical labelling of the different internal organs of the mother mouse and embryo. Multispectral imaging was achieved with fast sweeping of the laser wavelength on a per pulse basis within the NIR spectral window (wavelengths 700, 730, 755, 800 and 850 nm), which enabled rendering functional information from different foeto-maternal regions. Tissue excitation was carried out using a guided custom-made optical fibre bundle (CeramOptec GmbH, Bonn, Germany) bifurcated and terminated into 12 separate optical outputs placed at equi-angular distances at the top and bottom surface of the US transducer array. Each fibre-tip output provides a Gaussian illumination profile with a diameter of ~12 mm (full width at half maximum) at a distance of ~30 mm when immersed in water. The pressure (US) waves generated within the mice via thermal expansion were acquired using a ring array transducer (Imasonic SAS, France) covering a ~360˚ angular view around the imaged object. The ring array transducer consists of 512 adjacent piezoelectric sensors with a central detection frequency and −6 dB detection bandwidth of 5 MHz and ~80%, respectively. The individual array elements have a size of ~15 × 0.47 mm^2^ (height × width). The pressure waves for all elements were simultaneously sampled at 2032 instances with a sampling frequency of 40 MHz using a custom-made data acquisition system (Falkenstein Mikrosysteme GmbH, Taufkirchen, Germany). The acquired signals were subsequently transferred to a host PC through a 1 Gbit Ethernet connection for reconstruction and post-processing. The imaging system can acquire images at frame rates of up to 100 Hz determined by the PRF of the laser. Faster frame rates could be achieved with higher PRF lasers by sparsely acquiring signals from a reduced number of channels^41^.

### Animal preparation and in vivo imaging

All experimental procedures including animal handling were carried out in compliance with the institutional guidelines of Helmholtz Zentrum Muenchen and with the approval from the Government of Upper Bavaria. In this study, we used pregnant mice (CD-1 (Crl: CD1 ICR)) from Charles River, Wilmington, MA of two different days of gestation: E14 (*n* = 4) and E19 (*n* = 3). Pregnancy was safely determined by palpation of the embryos.

Prior to in vivo imaging, the abdominal region of the mice was thoroughly shaved in an anaesthetic environment with 1.2–2% v/v isoflurane in O_2_. Imaging was subsequently performed by placing each mouse in a specially designed holder (depicted in Supplementary Fig. 1) attached to the bottom of a water-filled tank. The mouse was kept stationary during the whole imaging process with its head outside the water. Isoflurane (1.2–2% v/v) in O_2_ or medical air was administered through a mouth clamp and gas anaesthesia breathing mask. The temperature of the water tank was adjusted to ~34 °C by using a feedback-controlled heating stick. In vivo imaging was initiated by laser radiation of 1064 nm with PRF of 25 Hz to capture anatomical 2D scans followed by multispectral recordings of the same cross-sections at five different laser wavelengths. A group of pregnant mice at different gestation days (E14: *n* = 2 and E19: *n* = 2) were imaged under a breathing gas challenge in which the inhaled gas was altered periodically between normoxia (medical air, 20% O_2_) and hyperoxia (100% O_2_) every 2 min for a total duration of 14 min. This periodic alteration of gasses was controlled manually using the gas flowmeters of the anaesthesia unit (UNO, Zevenaar, Netherlands) while keeping the isoflurane level constant. In a different experiment with an E14 pregnant mouse, 100 nmol of indocyanine green (ICG) diluted in 100 μl of phosphate buffer saline (PBS) was injected intravenously through a tail-vein catheter for 20 s. OA signals were acquired by tuning the laser to a wavelength of 800 nm and PRF of 25 Hz. It is also noted that all in vivo imaging experiments were terminal in the sense that all pregnant mice, including embryos, were euthanized during anaesthesia at the end of the experiments.

### Image reconstruction and multispectral unmixing

All acquired data were further analysed in MATLAB (vR2016b, Mathworks, Inc., MA, USA), installed in a graphics processing unit-based computer. A graphical user interface (GUI) was also developed to render on-the-fly image reconstruction (using a back-projection algorithm^42^) during each in vivo tomographic scan. The reconstruction parameters (e.g., speed of sound, image area, illumination wavelength, sampling instances, etc.) can be adjusted in the GUI for improved quality and better signal-to-noise ratio. Prior to image reconstruction, the time-resolved OA signals were deconvolved with the frequency response of the US transducer elements, followed by band-pass filtering (BPF) in the frequency range of 0.1 to 6 MHz. Each 2D cross-sectional scan of 45 × 45 mm^2^ was reconstructed in a 400 × 400 pixel area (pixel size of ~113 µm). Furthermore, all raw OA signals, corresponding to each scan, were also stored for offline image reconstruction, spectral unmixing and post-processing. The offline processing is described as follows. Following the aforementioned frequency-selective filtering, an adaptive filtering method was implemented based on least mean squares adaptation to reduce the noise effect within the frequency band of interest. This algorithm is described in detail in Section 1: Supplementary Document 1. Note that the adaptive filtering operation was executed simultaneously on all 512 signals for each tomographic scan. Next, the filtered signals were used for image reconstruction using a model-based approach^43,44^. The multispectral acquisitions were further unmixed to retrieve the distribution of oxygenated (HbO) and deoxygenated (HbR) haemoglobin^45^. This was achieved by least squares spectral fitting of individual pixels reconstructed at the five different wavelengths to the molar extinction spectra of HbO and HbR. Subsequently, pixel-wise oxygen saturation (SO_2_) mapping was calculated as SO_2_ = HbO/(HbO + HbR). The reconstructed images at 800 nm corresponding to the ICG injection experiment were additionally time-averaged for 10 consecutive frames to increase the signal to noise ratio (SNR).

### Post-processing of whole-body tomographic scans

After the reconstruction of whole-body anatomical cross-sections and multispectral unmixing, a few image processing steps were further implemented, which enabled the production of high-contrast OA images with substantial enhancement of subtle features that clearly demonstrate both structural and functional attributes in different experiments. First, the mouse surface in the 2D cross-sectional scans was segmented using a modified snake-based active contour algorithm^46^. The details of this processing step are presented in Section 2: Supplementary Document 1. The initial contour was labelled by manual segmentation of the surface, from which the algorithm converged to the actual boundary through an iterative approach. Both internal and external energy components were computed so that this progression would suffice. Next, a Gaussian smoothing operation was implemented on the segmented images to further reduce low frequency noise, followed by application of a high-pass unsharp mask to improve the contrast of high-frequency subtle structures present in the anatomical scans, thereby enhancing the visual appearance of the OA tomographic images.

## Supplementary information

Supplementary Material

Supplementary Video 1

Supplementary Video 2
