# Supplementary material for: Non-invasive determination of murine placental and foetal functional parameters with multispectral optoacoustic tomography
Source: Light Sci Appl. 2019 Aug 14;8:71. doi: 10.1038/s41377-019-0181-7 (PMC6804938; doi:10.1038/s41377-019-0181-7)
Supplement: Supplementary file 1 — Supplementary Material [file 41377_2019_181_MOESM1_ESM.docx]

Supplementary Information

Non-invasive determination of murine placental and foetal functional parameters with multispectral optoacoustic tomography

Kausik Basak^1,2,†^, Xosé Luís Deán-Ben^3,4^, Sven Gottschalk^2^, Michael Reiss^3,4^, Daniel Razansky^1,2,3,4,*^

^1^Faculty of Medicine, Technical University Munich, Germany

^2^Institute for Biological and Medical Imaging, Helmholtz Center Munich, Neuherberg, Germany

*^3^Faculty of Medicine and Institute of Pharmacology and Toxicology, University of Zurich, Switzerland*

*^4^Institute for Biomedical Engineering and Department of Information Technology and Electrical Engineering, ETH Zurich, Switzerland*

^*^Corresponding Author. E-mail: daniel.razansky@uzh.ch

^†^Present Address: Kausik Basak, Institute of Advanced Studies and Research, JIS University, Kolkata 700 091, West Bengal, India.

This file contains:

1. Supplementary Figures (with legends)
2. Supplementary Video Information
3. Supplementary Document

Supplementary Figures


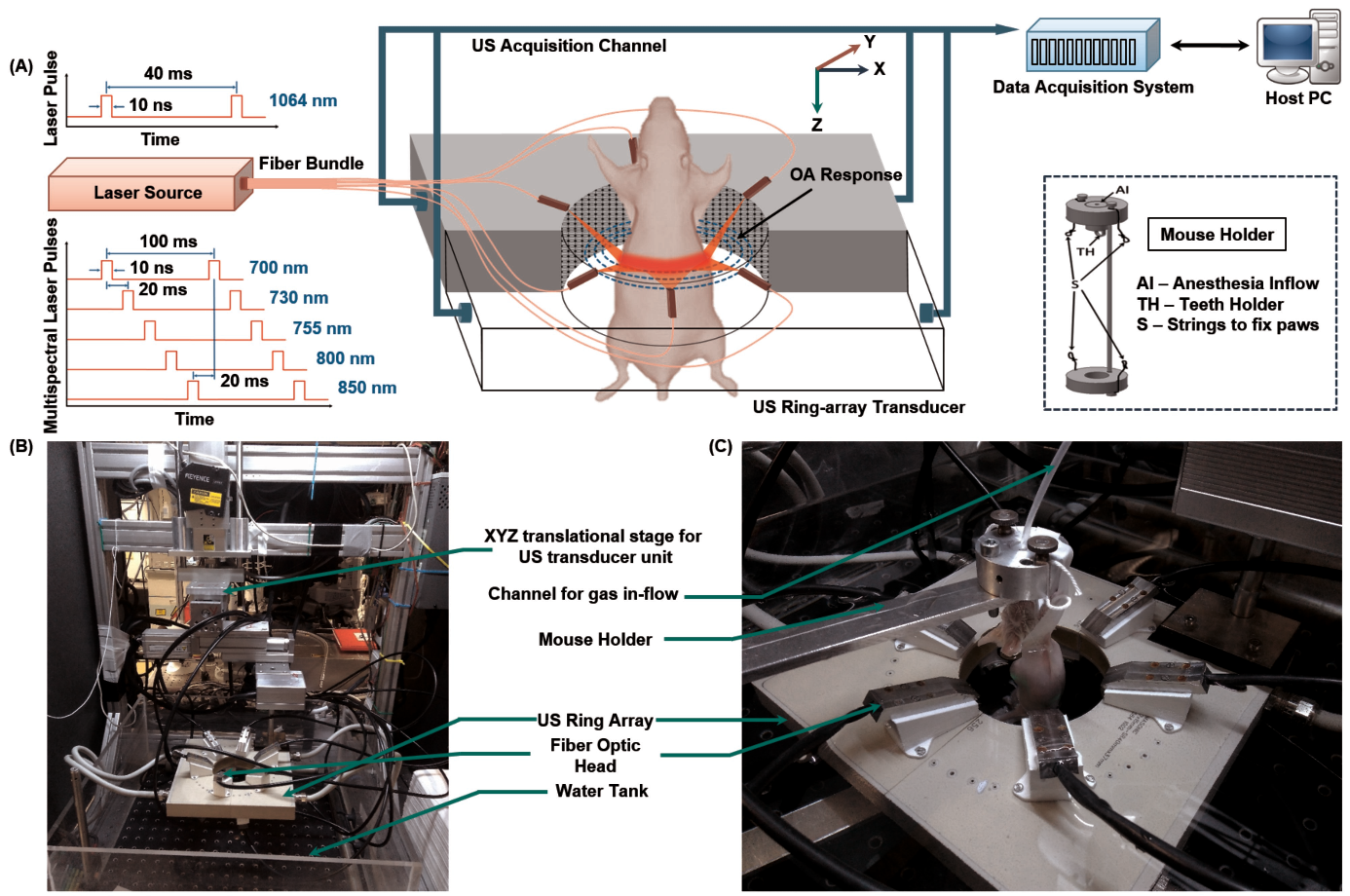


**Figure S1.** (A) Detailed imaging instrumentation of whole-body ring-shaped optoacoustic tomography (ROAT) system that facilitates *in vivo* and noninvasive imaging of mice with fast-scanning and high-resolution cross-sectional scans. At the inset, the schematic layout of mouse holder is provided along with its different parts. (B) Imaging instrumentation of ROAT system with its different parts and (C) mice holder and optical excitation with acoustic transder.


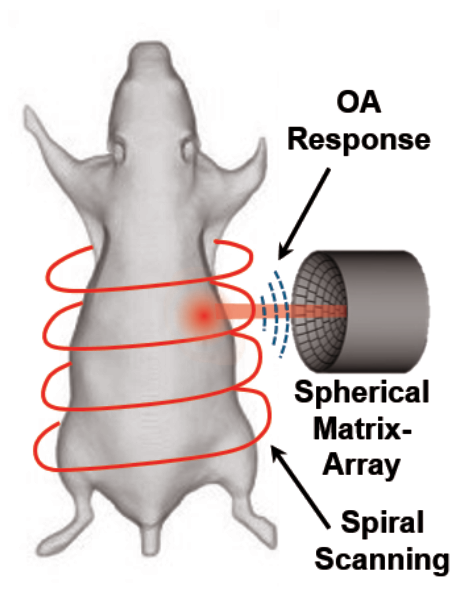


**Figure S2.** Lay-out of the spiral volumetric optoacoustic tomography (SVOT) imaging concept [10]. Whole-body tomographic data acquisition is performed along a spiral (helical) scanning trajectory by means of a spherical matrix ultrasound detection array. It takes about 5 minutes to acquire whole-body volumetric image data by combining all images acquired along the entire spiral trajectory.


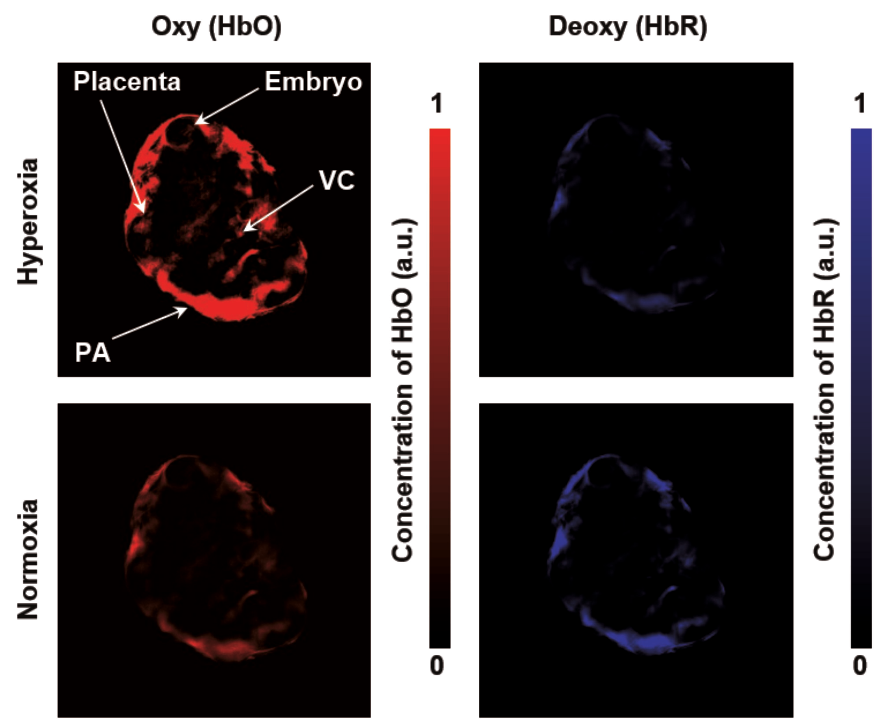


**Figure S3.** Spectrally unmixed images of HbO and HbR distributions in whole cross-section at hyperoxia and normoxia conditions. Main identifiable areas are labeled, including placenta, embryo, vena-cava (VC) and peripheral artery (PA) of the mother mouse.

Supplementary Video Information

Supplementary Video 1 : Fly-through movie of a vertical translation scan of E14 pregnant mouse.

Supplementary Video 2 : Fly-through movie of a reconstructed tomographic scans of E19 mouse.

Supplementary Document

**Section 1:**

**Filtering of OA Signals in Adaptive Domain: Least Mean Square Approach**

A degenerate form of adaptive noise canceller is implemented on band-pass filtered optoacoustic (OA) signals, simultaneously on 512 sensor elements, to reduce the background wideband noise. Its reference signal instead of being derived separately consists of a delayed version of the primary (input) signal. The delay is provided to de-correlate the noise signal so that the adaptive filter (used as a linear prediction filter) cannot predict the noise signal while easily predicting the signal of interest. Thus the output contains only the signal of interests which is again subtracted from the desired signal and the error signal is then used to adapt the filter weights to minimize the error. It adaptively filters the delayed version of the input signal in accordance with the least mean square (LMS) adaptation algorithm. The time domain computation of the ALE can be summarized as follows.

$x\left( n \right)=oa\left( n \right)+ noi\left( n \right)$ (1)

$r\left( n \right)= x(n-d)$ (2)

$y(n)= \sum_{k=0}^{L-1} w_{k}\left( n \right)r(n-k)$ (3)

$e\left( n \right)= x\left( n \right)-y(n)$ (4)

$w_{k}\left( n+1 \right)= w_{k}\left( n \right)+ \mu e\left( n \right)x(n-k-d)$ (5)

where $x\left( n \right)$ is the primary input signal corresponding to individual sensor element of ultrasound transducer array, consists of OA signal component [$oa\left( n \right)$] and wideband noise component [$noi\left( n \right)$]. The reference input signal $r\left( n \right)$ is the delayed version of the primary input signal by a delaying factor $d$. $y(n)$ is the output of the adaptive filter which is the best estimate of the desired response and $e\left( n \right)$ is the error signal at each iteration. $w_{k}\left( n \right)$ represents the adaptive filter weights and $L$ represents the adaptive filter length. The filter is selected as a linear combination of the past values of the reference input. There are three parameters that determine the performance of the algorithm for a given application.

A performance evaluation study of the proposed adaptive filtering approach is presented in SD Figure 1. The first column of the figure represents the raw vs. OA signals, acquired from different sensor elements at different time instances, clearly reflect the tracking capability and noise reduction of the adaptive algorithm. The estimated signals close track the changes in raw signals where subsequently diminishes the small flickering due to noisy effects which can be further justified through the analysis of power spectral density, laid out in second column of the figure. A significant reduction of signal power in the higher frequencies (> 8MHz) as well as efficient smoothing in the region 1 – 8 MHz is clearly visible, justifying the efficacy of the adaptive algorithm in reducing noisy artifacts. The third column signifies the mean square error (MSE) computation at every iteration of the filtering approach. At initial phase, the MSE is high due to time taken to adjust the step size parameter, after which a significant reduction in MSE, is observed at following iterations, which portrays a noteworthy performance of the adaptive approach in denoising the OA signals over time acquisition.


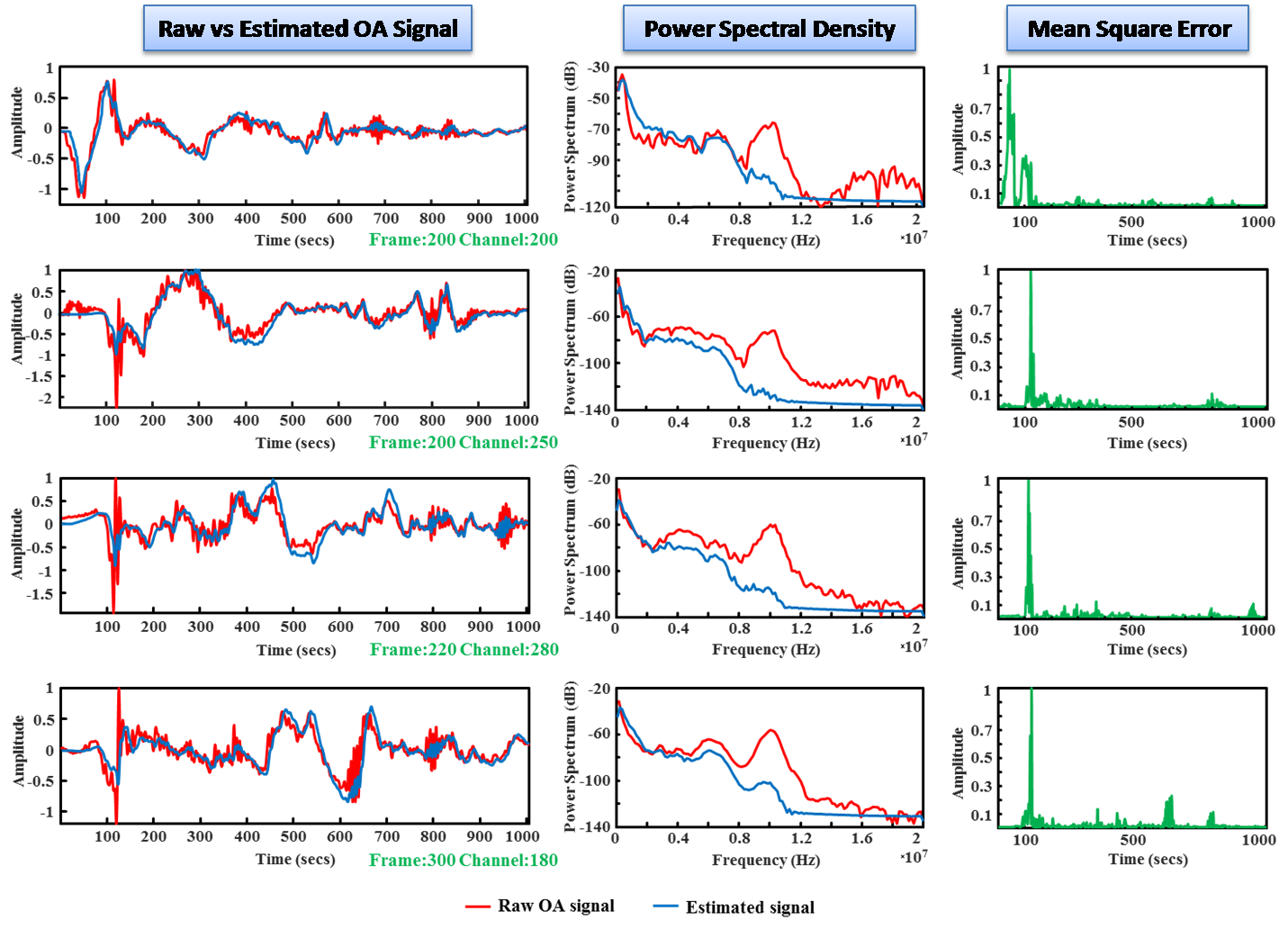


SD Figure 1. Performance evaluation of the proposed adaptive filtering algorithm. The first column represents raw vs. estimated OA signals for different sensor elements at different time instants, with their power spectral density is depicted in the second column. The third column stands for mean square error (MSE) performance of the proposed adaptive filtering approach.

**Section 2:**

**Segmentation of Pregnant Mice Area from OA reconstructed Images : Active Contour Implementation**

Segmentation of the pregnant mice area was performed using an improved snake based active contour (AC) method [Main Ref. 24]. The idea is to fit an energy-minimizing spline along the boundary, characterized by different internal and external image forces. The goal is to reach for a curve where the weighted sum of internal and external energy will be minimum. The basic equation can be formulated as,

$E_{snake}=\int_{0}^{1} \left\{E_{\mathrm{int}}\left(v\left(s\right)\right)+E_{ext}\left(v\left(s\right)\right)\right\}ds$ (6)

where, the position of snake is represented by a planar curve $v\left(s\right)=\left(x\left(s\right),y\left(s\right)\right)$,$E_{\mathrm{int}}$is the internal energy force, used to smooth the boundary during deformation.$E_{ext}$represents the external energies, pushing the snake towards the desired object. Seed contour for the initial labeling is performed using manual segmentation of the mice area. Coordinates of seed contour is transformed into polar form $\left(\rho,\theta\right)$. Quantization step for $\rho$and $\theta$are $\rho_{s}$and $\theta_{s}\left(=\frac{2\pi}{n}\right)$ respectively. The contour is now represented with a set of such discrete polar coordinates$v_{i}=\left(\rho_{i},\theta_{i}\right)\text{ for }i=0,1,2,....,\left(n-1\right)$; where$\theta_{i}=i\times\theta_{s}$. The centre is fixed at the centroid of seed contour. Quantization step size for angel $\theta$ is $\theta_{s}=1^{\circ}$(n = 360) and for $\rho$is $\rho_{s}$= 1 pixel. The energy function of this model is given by,

$E=\sum_{i=0}^{n-1} \left(aE_{cont}\left(v_{i}\right)+bE_{curv}\left(v_{i}\right)+cE_{image}\left(v_{i}\right)+dE_{grow}\left(v_{i}\right)\right)$ (7)





SD Figure 2. Active contour (snake) in polar coordinate

According to SD Figure 2, for each point$v_{i}\text{ for }i=0,1,2,....,\left(n-1\right)$, the energies at the points $\Omega_{i}=\left\{v_{i}^{-},v_{i},v_{i}^{+}\right\}$ are calculated and $v_{i}$ is moved to the point with the minimum energy among these three where $v_{i}^{-}$ and $v_{i}^{+}$ are the two discrete points adjacent to $v_{i}$at the radial direction. This operation is performed iteratively until the number of moved contour points is sufficiently small or the iteration time exceeds a predefined threshold. The weighting parameters of different energy functions are kept to be constants as *a* = 1, *b* = 0.8, *c* = *d* = 0.5. The length of radial direction (*R*) for computing image energy is chosen small (*R* = 3) for detecting sharp edges. The energy functions are: $E_{cont}$is the internal continuity spline energy that helps to maintain the contour to be continuous, $E_{curv}$is the internal curvature energy for smoothing the periphery,$E_{image}$is external image force that depends on the image intensity points and $E_{grow}$represents the external grow energy that helps to expand the contour from the centre towards the boundary. Mathematically they can be represented as [Main Ref. 24],

$E_{cont}\left(v_{j}\right)=\left|\bar{d}-\left|v_{j}-v_{i-1}\right|\right|+\left|\bar{\rho}-\left|\rho_{j}-\rho_{i-1}\right|\right|\text{ }\left(v_{j}\in\Omega_{i}\right)$ (8)

where, $\bar{d}=\sum\frac{\left|v_{t}-v_{t-1}\right|}{n}\text{ and }\bar{\rho}=\sum\frac{\left|\rho_{t}-\rho_{t-1}\right|}{n}$

$E_{curv}\left(v_{j}\right)=\left|v_{i+1}-2v_{j}+v_{i-1}\right|^{2}+\left|\rho_{i+1}-2\rho_{j}+\rho_{i-1}\right|^{2}\text{ }\left(v_{j}\in\Omega_{i}\right)$ (9)

$E_{image}\left(v_{j}\right)=\frac{1}{R}\sum_{r=1}^{R} I\left(\rho_{j}+r\times\rho_{s},\theta_{j}\right)-\frac{1}{R}\sum_{r=1}^{R} I\left(\rho_{j}-r\times\rho_{s},\theta_{j}\right)\text{ }\left(v_{j}\in\Omega_{i}\right)$ (10)

$E_{grow}\left(v_{j}\right)=\left\{\begin{matrix} e\text{ if v}_{j}=v_{i}^{+}\text{ and }\left|\bar{I}_{v_{j}}-\bar{I}_{origin}\right|<T \\ 0\text{ else} \end{matrix}\right\}$ (11)

where,$\bar{I}_{v_{j}}=\frac{1}{k\times k}\sum_{v_{i}\in\text{ }\Psi_{v_{j}}} I\left(v_{i}\right)\text{ and }\bar{I}_{origin}=\frac{1}{k\times k}\sum_{v_{i}\in\text{ }\Psi_{0}} I\left(v_{i}\right)\text{ }$

$\Psi_{v_{j}}$and $\Psi_{0}$are two $k\times k$($k$= 3) sub-blocks with centre points at $v_{i}$and the centroid of the contour respectively. The energy will decrease at $v_{i}^{+}$if both the sub-blocks are of same intensity approximately, resulting in an outward movement of the contour. This movement stops while the sub-blocks having different intensities. Threshold *T*(= 20) determines the range upto which the change in intensity is allowed. *e* is a negative constant (- 0.8), small value of which will limit the algorithm for more shape restrictions where large value of *e* also nullifies the effect of image energy for which the contour can exceed the actual boundary.
